# Passive and active suicidal ideation in a population-based sample of older adults: Associations with polygenic risk scores of relevance for suicidal behavior

**DOI:** 10.3389/fpsyt.2023.1101956

**Published:** 2023-02-21

**Authors:** Anna Zettergren, Mattias Jonson, Madeleine Mellqvist Fässberg, Jenna Najar, Therese Rydberg Sterner, Nazib M. Seidu, Silke Kern, Kaj Blennow, Henrik Zetterberg, Ingmar Skoog, Margda Waern

**Affiliations:** ^1^Neuropsychiatric Epidemiology Unit, Department of Psychiatry and Neurochemistry, Institute of Neuroscience and Physiology, the Sahlgrenska Academy, Centre for Ageing and Health (AGECAP) at the University of Gothenburg, Mölndal, Sweden; ^2^Region Västra Götaland, Sahlgrenska University Hospital, Psychiatry, Affective Clinic, Gothenburg, Sweden; ^3^Region Västra Götaland, Sahlgrenska University Hospital, Psychiatry, Cognition and Old Age Psychiatry Clinic, Gothenburg, Sweden; ^4^Aging Research Center, Department of Neurobiology, Care Sciences and Society, Karolinska Institutet and Stockholm University, Stockholm, Sweden; ^5^Department of Psychiatry and Neurochemistry, Institute of Neuroscience and Physiology, the Sahlgrenska Academy at the University of Gothenburg, Mölndal, Sweden; ^6^Clinical Neurochemistry Laboratory, Sahlgrenska University Hospital, Mölndal, Sweden; ^7^Department of Neurodegenerative Disease, UCL Institute of Neurology, London, United Kingdom; ^8^UK Dementia Research Institute at UCL, London, United Kingdom; ^9^Hong Kong Center for Neurodegenerative Diseases, Hong Kong, China; ^10^Region Västra Götaland, Sahlgrenska University Hospital, Psychosis Clinic, Mölndal, Sweden

**Keywords:** suicidal ideation, polygenic risk score, older adults, population-based sample, depression, neuroticism, cognitive performance

## Abstract

**Introduction:**

There are few studies investigating genetic factors related to suicidal ideation or behavior in older adult populations. Our aim was to test associations between passive and active suicidal ideation and polygenic risk scores (PRSs) for suicidality and other traits of relevance for suicidality in old age (i.e. depression, neuroticism, loneliness, Alzheimer’s disease, cognitive performance, educational attainment, and several specified vascular diseases) in a population-based sample aged 70 years and older.

**Methods:**

Participants in the prospective H70 study in Gothenburg, Sweden, took part in a psychiatric examination that included the Paykel questions on active and passive suicidal ideation. Genotyping was performed with the Neurochip (Illumina). After quality control of the genetic data the sample included 3467 participants. PRSs for suicidality and other related traits were calculated based on summary statistics from recent GWASs of relevance. Exclusion of persons with dementia or incomplete data on suicidal ideation yielded 3019 participants, age range 70–101 years. Associations between past year suicidal ideation (any level) and selected PRSs were analysed using general estimation equation (GEE) models, adjusted for sex and age.

**Results:**

We observed associations between passive/active suicidal ideation and PRSs for depression (three versions), neuroticism, and general cognitive performance. After excluding individuals with current major depressive disorder (MDD), similar associations were seen with PRS for neuroticism, general cognitive performance and two PRSs for depression. No associations were found between suicidal ideation and PRSs for suicidality, loneliness, Alzheimer’s disease, educational attainment, or vascular disease.

**Discussion:**

Our results could indicate which types of genetic susceptibility that are of importance for suicidality in old age, and these findings can help to shed light on potential mechanisms that may be involved in passive and active suicidal ideation in late-life, also in those with no current MDD. However, due to the limited sample size, the results need to be interpreted with caution until replicated in larger samples.

## 1. Introduction

In most parts of the world, older adults have the highest suicide rates (1). The prevalence of past year passive and active suicidal ideation in individuals between the ages of 70 and 108 has been reported to be 11%, with a considerably higher percentage (37%) among people with depression (2). Suicidal ideation can signal the start of a suicidal process. Almost three quarters of older adults who died by suicide had reported some level of passive or active suicidal ideation to their next of kin during their last year of life (3).

Results from twin studies have provided evidence for a genetic component in suicidal ideation and behavior, with heredity estimates of around 30–55% (4). Candidate gene studies of suicidality have mainly focused on genes related to neurotransmitter signaling, but few findings have been consistent, and the highlighted genes have not been confirmed in genome-wide association studies (GWASs) (5). Overall, GWASs of suicidality have reported a very limited number of genetic markers reaching the stringent level of genome-wide significance (6–16). This might be explained by limited sample size, designs with other primary outcomes, and diagnostic heterogeneity. Moreover, many of these studies were performed within psychiatric samples with severe mental illness. Recent GWASs have identified some genome-wide significant associations with suicide attempt (17), as well as a broad suicidality phenotype (18). So far, studies investigating genetic factors related to suicidal behavior or ideation specifically in old populations are very sparse (19).

Suicidality in older adults is associated with a range of psychiatric and somatic factors, but also social situation. The most robust association is that with depressive disorder (20, 21), but also anxiety has been associated with suicidal passive/active ideation in older adults (22). Somatic factors associated with suicidality in older adults are, for example: functional disability (23), heart disease (24), and stroke (25). Regarding social factors, low educational level (26), interpersonal conflicts, and loneliness (27) have been highlighted. Other factors reported to be associated with suicidality in old age are personality [i.e., neuroticism (28)] and cognitive impairment (29). The heterogeneity of suicidality in old age has created problems for those who attempt to identify biological markers. One way forward is to study associations with genetic profiles (i.e., polygenic risk scores) of different traits of relevance for suicidal ideation and behavior. Although PRSs within psychiatry are not able to definitely predict a diagnosis, they can contribute to risk assessment in combination with information from clinical and lifestyle metrics (30). The aim of the present study was to test associations between polygenic risk scores (PRSs) for suicidality, but also for other traits of relevance for suicidality in old age (i.e., depression, neuroticism, loneliness, Alzheimer’s disease, cognitive performance, educational attainment, and vascular diseases), and passive and active suicidal ideation in a population-based sample aged 70 years and older. Summary statistics from recent GWASs highlighting genes involved in CNS development, synaptic structure and neurotransmission (18, 31–38), gene regulation and neurodegeneration (17, 33, 36, 39, 40), metabolic pathways (40), and vascular mechanisms (41) were used for construction of the PRSs.

## 2. Methods

### 2.1. Study population

Participants in the study were recruited in connection with two population-based epidemiological studies in Gothenburg, the Prospective Population Study of Women (PPSW) and the Gothenburg Birth Cohort Studies (H70, H85 and 95+), described in detail previously (42–45).

**Figure 1:**
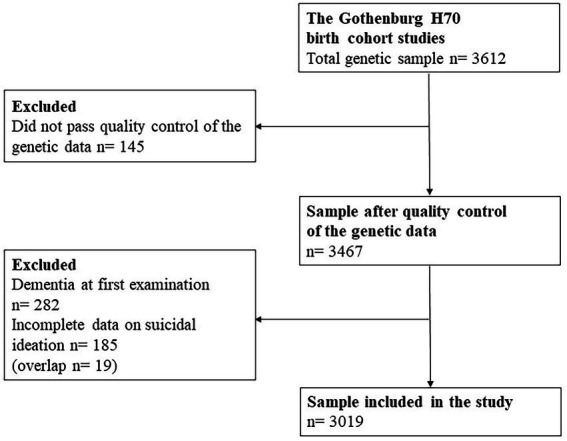
Flow diagram of the study population.

Genetic data were available for a total of 3,612 individuals. After quality control of the genetic data (for description of the quality control see section below about genotyping), 3,467 out of these 3,612 remained. Exclusion of persons with dementia at the time of their first examination (*n* = 282) or incomplete data on suicidal ideation (*n* = 185) yielded a total of 3,019 participants for the current study (Figure 1), age range 70–101 years.

The study was approved by the Regional Ethical Review Boards in Gothenburg. Informed consent was obtained from all participants and/or their relatives in cases of dementia.

### 2.2. Examinations and diagnoses

The interview included the Paykel questions on passive and active suicidal ideation and behavior (46). These questions, originally dubbed “suicidal feelings,” have been applied in numerous population-based studies involving older adults [see (2) for an overview]. Four items cover ideation of increasing intensity; a fifth involves an actual suicide attempt: (1) “Have you ever felt that life was not worth living?” (2), “Have you ever wished you were dead?,” (3) Have you ever thought of taking your life, even if you would not really do it?” (4) “Have you ever reached the point where you seriously considered taking your life, or perhaps made plans how you would go about doing it?,” and (5) “Have you ever made an attempt to take your life?.” As previously reported, inter-rater reliability ranged 0.96 (life-weariness) to 0.74 (seriously considered taking life) (2). Participants with affirmative responses were asked to report the most recent occurrence of these feelings, and within the current study individuals who reported “yes” on any Paykel question, with the occurrence “past year,” were considered as “cases.”

Dementia and major depressive disorder (MDD) were diagnosed by geriatric psychiatrists. A computerized algorithm based on The Comprehensive Psychopathological Rating Scale (CPRS) items was employed to establish whether participants met diagnostic criteria for MDD (47). The algorithm was designed to follow the definition of major depressive episode in the Diagnostic and Statistical Manual for Mental Disorders, Fifth Edition (DSM-5) (48) as closely as possible: At least five of the nine depressive symptoms or signs listed in DSM-5 had to be present, at least one of which had to be either depressed mood or loss of interest. Exclusion criteria for depression could not be applied due to the difficulty of making etiological judgments in an epidemiological study. Dementia was diagnosed according to the Diagnostic and Statistical Manual of Mental Disorders 3rd Edition Revised (DSM-III-R) (49).

### 2.3. Genotyping

Genotyping was performed with the NeuroChip (Illumina) (50). Quality control (QC), described previously (51, 52), included the removal of individuals due to any of the following: per-individual call rate < 98%, sex mismatch, and excessive heterozygosity (FHET outside +/−0.2). Further, individuals were defined as non-European ancestral outliers, and removed, if their first two PCs exceeded 6 standard deviations from the mean values of the European samples in the 1,000 Genome global reference population. Closely related individuals were removed based on pairwise PI_HAT (i.e., proportion of the genome that is in identity-by-descent; calculated using genome option in PLINK) > =0.2. Genetic variants were excluded due to: per-SNP call rate < 98%, minor allele frequency (MAF) <0.01, and Hardy–Weinberg disequilibrium (*p* < 1e-6). The Sanger imputation service (53) was used to impute post-QC, using the reference panel of Haplotype Reference Consortium data (HRC1.1).

### 2.4. Construction of polygenic risk scores

PRSs were constructed based on summary statistics from previous GWASs, available through the IEU GWAS database (54) and the GWAS central database (55); for further details see Supplementary Table 1. SNPs with MAF ≥5% were used for selection by linkage disequilibrium (LD)-clumping. The European ancestry samples from the 1,000-genomes project were used as a reference panel to remove variants in LD; all variants 250 kb upstream and downstream of top signal were removed (*R*^2^ < 0.001). All PRSs were calculated as the sum of the β-coefficient multiplied with the number of effect alleles of each genetic variant, and then standardized. We created PRSs including variants based on the GWAS *p*-value thresholds *p* < 5e^−8^ and *p* < 1e^−5^ (referred to as 5e^−8^ PRS and 1e^−5^ PRS) if the summary statistics were retrieved from the IEU GWAS database, and on the *p* < 5e^−8^ level if the summary statistics came from the GWAS central database. The PRS for Alzheimer’s disease (AD-PRS) (on the *p* < 5e^−8^ and *p* < 1e^−5^ thresholds) was based on GWAS summary statistics in the IGAP (56), and PRSs for suicidality and suicide attempt (on the *p* < 5e^−8^ and *p* < 1e^−6^ thresholds, respectively) were based on summary statistics presented within the GWASs by Erlangsen et al. (17) and Strawbridge et al. (18). The suicidality phenotype used in the study by Strawbridge et al. (18) is a measure of suicidality based on the categories “no suicidality,” “thoughts that life was not worth living,” “ever contemplated self-harm or suicide,” “acts of deliberate self-harm not including attempted suicide,” and “attempted suicide.”

### 2.5. Statistical analyses

Sample characteristics were compared using Fisher’s exact test (categorical variables) or *t*-test (continuous variables). Associations between PRSs and past year passive/active suicidal ideation were investigated using general estimation equation (GEE) adjusted for age, sex, and 10 principal components (PCs) to correct for population stratification. The use of GEE analyses enables repeated measurements to be taken into account, and data based on examinations when individuals fulfilled the criteria for a dementia diagnosis were excluded. All analyses were then repeated after also excluding data based on examinations when individuals were diagnosed with MDD. Further, sensitivity analyses were performed based on passive suicidal ideation only (Paykel questions 1 and 2). Correction for multiple testing was performed using the Bonferroni method. Due to overlap between many of the PRSs, all tests performed could not be considered independent of each other, and we therefore corrected for the number of domains included, i.e., psychiatric conditions (depression and suicidality), personality (neuroticism), cognitive function/performance (general cognitive performance, Alzheimer’s disease, educational attainment), loneliness, and vascular disease (stroke, hypertension, atherosclerotic heart disease, and angina), generating a corrected *p*-value threshold of *p* = 0.01. The statistical analyses were performed in IBM SPSS Statistics v28.

## 3. Results

Characteristics of the study sample are presented in Table 1. Individuals who had ever answered with an affirmative response on any of the Paykel questions on past year passive/active suicidal ideation (33% of the total sample) were more often women, and more often fulfilled the criteria for dementia and MDD. They were also significantly older at their first examination and had participated in more examinations than those who never answered yes.

**Table 1:** Sample characteristics.

	Suicidal feelings (*n* = 440)	No suicidal feelings (*n* = 2,579)	*p*-value
Sex: women, n (%)	354 (80.5)	1,568 (60.8)	<0.001
Age at first exam, mean (sd)	78.0 (8.8)	75.2 (7.6)	<0.001
Developed dementia, n (%)	118 (26.8)	309 (12.0)	<0.001
Major depression ever, n (%)	165 (37.5)	85 (3.3)	<0.001
Number of exams*, mean (sd)	2.6 (1.4)	1.8 (1.1)	<0.001

*Only examinations with data on Paykel questions.

Associations were observed between past year active/passive suicidal ideation and three versions of PRSs for MDD. We also found association with a broad depression phenotype defined as “self-reported past help-seeking for problems with ‘nerves, anxiety, tension or depression’.” In addition, passive/active suicidal ideation was associated with PRSs for neuroticism (two versions based on different GWASs), and general cognitive performance/ability (higher score on the PRS for general cognitive performance was associated with decreased risk for ideation; Table 2 and Supplementary Figure 1). Associations remained after Bonferroni correction for multiple testing for all but two PRSs (one PRS for MDD and one for neuroticism). After excluding individuals with current MDD, similar associations were seen with PRSs for neuroticism, general cognitive performance, and three PRSs for MDD (Table 2 and Supplementary Figure 1). After correction for multiple testing associations with two depression-PRSs, one neuroticism-PRS, and the PRS for cognitive performance remained.

**Table 2:** Associations between polygenic risk scores and suicidal feelings in a population-based sample of older adults (*n* = 3,019).

			Major depression included					Major depression excluded				
		GWAS-level (n: SNPs)			95% CI					95% CI		
Trait	Score-id		Beta	SE	Lower	Upper	*p*-value	Beta	SE	Lower	Upper	*p*-value
Suicide attempts	Erlangsen_2020	1e-6 (n: 4)	−0.049	0.0526	−0.152	0.054	0.4	−0.060	0.0613	−0.180	0.061	0.3
Suicidality broad	Strawbridge_2019	5e-8 (n: 3)	0.059	0.0563	−0.051	0.169	0.3	0.066	0.0655	−0.062	0.195	0.3
Depression	gwc-GCST007342	5e-8 (n: 69)	0.171	0.0588	0.056	0.286	0.004*	0.182	0.0673	0.050	0.314	0.007*
Major depressive disorder	gwc-GCST006041	5e-8 (n: 11)	0.156	0.0529	0.052	0.259	0.003*	0.155	0.0579	0.042	0.269	0.007*
Depressive symptoms	gwc-GCST007340	5e-8 (n: 137)	0.114	0.0595	−0.003	0.231	0.06	0.111	0.0683	−0.023	0.245	0.1
Depression (broad)	ebi-a-GCST005902	1e-5 (n: 167)	0.175	0.0692	0.039	0.310	0.01*	0.137	0.0800	−0.020	0.294	0.09
Depression (broad)	ebi-a-GCST005902	5e-8 (n: 22)	0.079	0.1024	−0.122	0.280	0.4	0.100	0.1171	−0.130	0.329	0.4
Major depressive disorder	ieu-a-1,187	1e-5 (n: 201)	0.069	0.0569	−0.042	0.181	0.2	0.051	0.0643	−0.075	0.177	0.4
Major depressive disorder	ieu-a-1,187	5e-8 (n: 39)	0.114	0.0555	0.005	0.223	**0.04**	0.145	0.0648	0.018	0.272	**0.03**
Depression ever diagnosed	ukb-d-20544_11	1e-5 (n: 40)	0.038	0.0884	−0.136	0.211	0.7	0.098	0.1006	−0.099	0.295	0.3
Depression ever diagnosed	ukb-d-20544_11	5e-8 (n: 6)	−0.031	0.1156	−0.257	0.196	0.8	0.068	0.1341	−0.195	0.331	0.6
Alzheimer’s disease	Kunkle_2019	1e-5 (n: 66)	−0.090	0.0611	−0.210	0.029	0.1	−0.083	0.0699	−0.220	0.054	0.2
Alzheimer’s disease	Kunkle_2019	5e-8 (n: 25)	−0.073	0.0628	−0.196	0.050	0.2	−0.055	0.0715	−0.195	0.085	0.4
Cognitive performance	ebi-a-GCST006572	1e-5 (n: 680)	−0.199	0.0580	−0.312	−0.085	0.0006*	−0.221	0.0655	−0.349	−0.093	0.0007*
Cognitive performance	ebi-a-GCST006572	5e-8 (n: 205)	−0.171	0.0586	−0.286	−0.056	0.004*	−0.168	0.0672	−0.299	−0.036	0.01*
Educational attainment	gwc-GCST006442	5e-8 (n: 498)	−0.008	0.0527	−0.111	0.095	0.9	0.017	0.0595	−0.100	0.133	0.8
Neuroticism	ebi-a-GCST006940	1e-5 (n: 472)	0.154	0.0566	0.043	0.265	0.006*	0.149	0.0614	0.028	0.269	**0.02**
Neuroticism	ebi-a-GCST006940	5e-8 (n: 114)	0.141	0.0550	0.033	0.248	0.01*	0.171	0.0607	0.052	0.290	0.005*
Neuroticism	ebi-a-GCST005232	1e-5 (n: 393)	0.117	0.0648	−0.010	0.244	0.07	0.122	0.0734	−0.022	0.266	0.1
Neuroticism	ebi-a-GCST005232	5e-8 (n: 94)	0.111	0.0729	−0.031	0.254	0.1	0.155	0.0829	−0.007	0.317	0.06
Neuroticism score	ukb-b-4,630	1e-5 (n: 530)	0.128	0.0600	0.011	0.246	**0.03**	0.104	0.0659	−0.025	0.233	0.1
Neuroticism score	ukb-b-4,630	5e-8 (n: 145)	0.110	0.0642	−0.016	0.236	0.09	0.146	0.0711	0.007	0.286	**0.04**
Loneliness, isolation	ukb-b-8,476	1e-5 (n: 123)	0.029	0.0535	−0.076	0.134	0.6	0.018	0.0632	−0.106	0.142	0.8
Loneliness, isolation	ukb-b-8,476	5e-8 (n: 17)	−0.058	0.0526	−0.161	0.045	0.3	−0.054	0.0623	−0.176	0.068	0.4
Feeling lonely	ebi-a-GCST006942	1e-5 (n: 87)	0.070	0.0522	−0.033	0.172	0.2	0.072	0.0598	−0.046	0.189	0.2
Feeling lonely	ebi-a-GCST006942	5e-8 (n: 7)	0.024	0.0524	−0.078	0.127	0.6	0.007	0.0594	−0.110	0.123	0.9
Loneliness	gwc-GCST006923	5e-8 (n: 9)	−0.031	0.0526	−0.134	0.072	0.6	−0.047	0.0600	−0.165	0.070	0.4
Ischemic stroke	ebi-a-GCST005843	1e-5 (n: 90)	−0.024	0.0544	−0.131	0.083	0.7	0.019	0.0628	−0.104	0.142	0.8
Ischemic stroke	ebi-a-GCST005843	5e-8 (n: 18)	0.002	0.0530	−0.102	0.106	0.99	0.047	0.0610	−0.073	0.166	0.4
hypertension	ukb-b-12,493	1e-5 (n: 289)	−0.023	0.0585	−0.138	0.091	0.7	0.011	0.0670	−0.121	0.142	0.9
hypertension	ukb-b-12,493	5e-8 (n: 80)	0.005	0.0545	−0.102	0.112	0.9	0.088	0.0621	−0.034	0.210	0.2
High blood pressure	ukb-b-14,177	1e-5 (n: 769)	0.073	0.0573	−0.039	0.185	0.2	0.111	0.0668	−0.020	0.241	0.1
High blood pressure	ukb-b-14,177	5e-8 (n: 331)	0.019	0.0580	−0.095	0.132	0.7	0.031	0.0667	−0.100	0.162	0.6
Atheroscelrotic heart disease	ukb-b-1,668	1e-5 (n: 108)	−0.027	0.0519	−0.128	0.075	0.6	0.027	0.0585	−0.087	0.142	0.6
Atheroscelrotic heart disease	ukb-b-1,668	5e-8 (n: 30)	−0.009	0.0547	−0.116	0.098	0.9	0.053	0.0624	−0.069	0.176	0.4
Angina	ukb-b-8,468	1e-5 (n: 91)	0.075	0.0554	−0.033	0.184	0.2	0.093	0.0637	−0.032	0.218	0.1
Angina	ukb-b-8,468	5e-8 (n: 23)	0.071	0.0542	−0.035	0.177	0.2	0.112	0.0633	−0.012	0.236	0.07

Significant value of *p*s at the level 0.05 are shown in bold. *Value of *p*s significant after Bonferroni correction for multiple testing (corrected *p*-value threshold = 0.01; based on testing of PRSs within five different domains related to suicidal feelings in old age: psychiatric disease, personality, cognitive function/performance, loneliness, vascular disease.

No associations were found in relation to PRSs for neither suicide attempts nor the broad suicidality phenotype that included passive ideation. Further, no associations were found for PRSs for loneliness, Alzheimer’s disease, educational attainment, and vascular diseases (stroke, hypertension, atherosclerotic heart disease, and angina; Table 2 and Supplementary Figure 1).

Analyses based on passive suicidal ideation only (i.e., life not worth living, death wishes) generated results similar to those based on all five Paykel questions, with some exceptions. Some minor differences were found regarding PRSs surviving correction for multiple testing, as well as a weak association, not surviving correction, with PRS for angina in the analysis excluding individuals with current MDD (Supplementary Table 2).

## 4. Discussion

To the best of our knowledge, this is the first study to investigate polygenic risk scores for suicidality, and traits of potential relevance for suicidality in old age, in relation to passive and active suicidal ideation in a population-based sample of older adults. Associations were found with PRSs for depression, neuroticism, and cognitive performance, while no associations were seen with PRS for suicidality, loneliness, educational attainment, Alzheimer’s disease, and several specified vascular diseases.

Among the genetic risk profiles included in this study, PRS for depression was highly expected to be associated with passive/active suicidal ideation due to the robust association between depression and suicidality in old age (20, 21). Suicidal ideation is one of the nine criteria for MDD (DSM-5) (48). In total, relationships with six PRSs for depression were examined, and associations were found with four of these (three after correction for multiple testing). Although there is an overlap among PRSs, this indicates a stable association between genetic risk for depression and passive/active suicidal ideation in our cohort of older individuals. After excluding individuals with current MDD, associations with three of the PRSs (those for MDD) still remained (two after correction for multiple testing). Our result expands on findings from a previous study on younger individuals, showing an association between a PRS for MDD and suicide attempt (10). That study was however performed within a psychiatric sample, including individuals with MDD, bipolar disorder, and schizophrenia. Considering the whole genome, the strongest genetic correlation between suicidality and major psychiatric disorders in a population-based sample of individuals aged 37–73 was seen with MDD in the study that applied the phenotype that included passive ideation (18).

In our study, we also found associations between passive/active suicidal ideation in later life and two of three included PRSs for neuroticism. Similar to the case with MDD, genetic correlation between suicidality and neuroticism has been demonstrated in younger age groups (18). The association between suicidal ideation and one of the PRSs for neuroticism remained also after excluding individuals with MDD. This contrasts somewhat with our clinical cohort study in which the association between neuroticism and suicide attempt disappeared after adjusting for MDD (28). Previous studies have shown a genetic overlap between MDD and neuroticism (31, 33), but the overlap between specific SNPs in the PRSs for MDD and neuroticism used in our study was limited.

We also found an association between PRS for general cognitive performance and passive/active suicidal ideation. This parallels a finding reported in a Mendelian randomization study of the genetic influence of cognitive performance and educational attainment on suicidal attempt risk (57). However, an association was also observed with educational attainment in that study, which was not the case in ours. Further, educational attainment was found to drive the association between cognitive performance and suicide attempt risk. The PRSs for cognitive performance and educational attainment used in our study were based on GWAS summary statistics from studies on cohorts with a mean year of birth between 1936 and 1979 (37). It is possible that the PRS for educational attainment is not as representative for a sample born 1901–1944, as the PRS for cognitive performance. Moreover, compromised cognitive performance in older adults is different from compromised cognitive decline in younger persons, as it may be the result of cognitive decline rather than a marker for lower educational level.

Given recent findings of cognitive deficits (29, 58, 59), as well as risk of subsequent dementia (60) in suicidal older adults, we anticipated an association with the AD-PRS. Moreover, a previous study of relationships between suicidal ideation and biopsychosocial predictors in old age reported a trend toward an association with carriership of the genetic AD risk factor *APOE ε4* (19). This genetic risk factor is a strong contributor within the AD-PRS used in our study. However, no association with the AD-PRS was seen. One explanation could be that individuals with dementia (i.e., including those with highest level of the AD-PRS) were excluded from the study. However, individuals with high risk of dementia were not completely left out, since several individuals within the continuum of MCI to “just below the threshold of a dementia diagnosis” were included (61). Further, although we found an association with PRS for cognitive performance, this PRS shows very limited overlap with the AD-PRS, and the scores have been reported to associate differently with cognitive decline in normal aging (62).

We found no association between PRSs for suicidality and passive/active suicidal ideation in old age. Two different PRSs for suicidality, based on the most recent GWASs, were tested; one for suicide attempts (17) and one for a broad suicidality phenotype (18). Neither PRS can be considered to measure suicidality exactly as it is defined in the present study, and genetics behind different suicidal phenomena (i.e., ideation, attempt, and suicide) might differ to some extent. For example, a PRSs for suicide attempt (based on GWASs using lifetime data on suicide attempts during depressive episodes) did not predict suicidal ideation in a previous study (16), which is in line with epidemiological studies, showing that suicidal ideators/attempters/completers differ in sociodemographic characteristics (63, 64). In the current study, results for passive suicidal ideation did not differ from findings of the analyses that included both passive and active ideation. However, it must be stressed that our study is population-based and very few participants reported past year suicide attempts.

We found no associations with PRSs for loneliness and several vascular diseases, indicating that genetic factors behind these traits are not a major driving force when it comes to genetic susceptibility for passive/active suicidal ideation in later life. It might be that either the influences of these PRSs on such ideation are too small to be detected in our sample, or the relations between loneliness, vascular disease, and suicidal passive/active ideation are explained primarily by environmental and lifestyle factor components of these traits. Still, some SNPs in the PRSs for loneliness overlap with the PRSs for neuroticism found to be associated with suicidal ideation in our study and increasing the sample size might be a way to detect an association between PRS for loneliness and suicidal ideation.

Strengths with this study are the well-characterized participants, the relative homogeneity of the sample, and the population-based setting that is systematically selected in order to be representative for older individuals in the general population (42).

There are however some limitations of the study. The population-based setting means that few participants had active suicidal ideation. Further, the sample is relatively small for a genetic study, which prevents sub-group analyses based on sex and age, or age of ideation onset. The latter is of interest considering the possibility of differential etiologies of early and late onset suicidal behavior in older adults (58, 65). The small sample size also means that although the power to detect genetic findings can to some extent be increased by combining genetic signals into PRSs, the current study can still be underpowered to detect associations. This applies above all to the PRSs that are based on summary statistics from very large discovery samples, since the SNPs originating from such samples often contributes with very small effects or might be too rare to contribute with any impact in our study. The findings in our study remained similar after excluding individual’s examination times with current MDD. However, a stricter exclusion, based on for instance life time expression, might have changed the results. Due to the limited sample size and limited information about life time depression, these types of analyses were not possible to perform within the frame of our study.

Since full summary statistics were not always available, the PRSs used in the study were selected based on pre-defined *p*-value thresholds, and in some cases a more optimal *p*-value threshold might have strengthened the result. In addition, novel GWASs are published continuously, probably increasing the predictive value of PRSs for the different traits used in this study. Since novel GWASs often are larger than their predecessors, they will very likely generate additional SNPs to include in PRSs. Moreover, the discovery cohorts used in the GWASs that were bases for the PRSs do overlap (e.g., samples from the UK Biobank), but importantly, there is no overlap between the discovery and target samples, which might otherwise have caused overfitting due to non-independency of the discovery and target data. Finally, most participants were of Scandinavian descent and although sample homogeneity is a strength in genetic association studies, the results are not generalizable to other ethnic groups.

In conclusion, this study reports PRSs for depression, neuroticism, and cognitive performance to be associated with passive/active suicidal ideation among older individuals in a population-based sample, both before and after excluding current MDD. There were however no associations seen when analyzing PRSs for suicidality, loneliness, educational attainment, Alzheimer’s disease, and vascular disease. Our results could indicate which types of genetic susceptibility that are of importance for suicidality in old age, and these findings can help to shed light on potential mechanisms that may be involved in passive and active suicidal ideation in late-life, also in those with no current MDD. Considering the type of study, the sample size is somewhat limited, and results must be interpreted with caution until replicated in larger samples.

## Data availability statement

The datasets used for this study is available upon request through https://www.gu.se/en/research/epinep/ and https://www.near-aging.se/databases/.

## Ethics statement

The studies involving human participants were reviewed and approved by the Regional Ethical Review Boards in Gothenburg. The patients/participants provided their written informed consent to participate in this study.

## Author contributions

AZ, MJ, MF, and MW designed the study. AZ, MJ, MF, JN, TR, NS, IS, SK, and MW took part in the acquisition of subjects and data. AZ analyzed the data. AZ, MJ, and MW took part in the interpretation of the data, drafted the manuscript, and all the other authors revised it critically for important intellectual content. AZ, SK, HZ, KB, and MW funded the study. All authors approved the final version of the manuscript.

## Funding

This study was supported by grants from the Swedish Research Council (2012–5041, 2013–8717, 2015–02830, 2016–01590, 2017–00639, 2018–02532, 2019–01096, 2019–02075), the Swedish Research Council for Health, Working Life and Welfare (Forte) (2013–1202, AGECAP 2013–2300, 2013–2496, 2016–07097, 2018–00471), the European Research Council (681712 and 101053962), Swedish State Support for Clinical Research (ALFGBG-71320, ALFGBG-965923, ALFGBG-81392, ALFGBG-771071, ALFGBG-715-841, ALFGBG-965-525, ALFGBG-716681), the Alzheimer Drug Discovery Foundation (ADDF), United States (201809–2016862), the AD Strategic Fund and the Alzheimer’s Association (ADSF-21-831376-C, ADSF-21-831381-C, and ADSF-21-831377-C), the Bluefield Project, the Olav Thon Foundation, the Erling-Persson Family Foundation, Stiftelsen för Gamla Tjänarinnor, Hjärnfonden (FO2022-0270, FO2014-0207, FO2016-0214, FO2018-0214, FO2019-0163), the European Union’s Horizon 2020 Research and Innovation Programme under the Marie Skłodowska-Curie grant agreement No 860197 (MIRIADE), the European Union Joint Programme—Neurodegenerative Disease Research (JPND2021-00694), and the UK Dementia Research Institute at UCL (UKDRI-1003), Alzheimerfonden (AF-842471, AF-737641, AF-929959, AF-939825, AF-554461, AF-647651, AF-743701, AF-844671, AF-930868, AF-940139, AF968431, AF-939988, AF-930582), Psykiatriska Forskningsfonden, Stiftelsen Demensfonden, Stiftelsen Hjalmar Svenssons Forskningsfond, Stiftelsen Wilhelm och Martina Lundgrens vetenskapsfond, Konung Gustaf V:s och Drottning Victorias Frimurarestiftelse and Agneta Prytz-Folkes och Gösta Folke’s Foundation.

## Conflict of interest

SK has been consultant for Geras Solutions, unrelated to the findings in this manuscript. HZ has served at scientific advisory boards for Eisai, Denali, Roche Diagnostics, Wave, Samumed, Siemens Healthineers, Pinteon Therapeutics, Nervgen, AZTherapies, and CogRx, has given lectures in symposia sponsored by Cellectricon, Fujirebio, Alzecure, and Biogen, and is a co-founder of Brain Biomarker Solutions in Gothenburg AB (BBS), which is a part of the GU Ventures Incubator Program (outside submitted work). KB has served as a consultant, at advisory boards, or at data monitoring committees for Abcam, Axon, Biogen, JOMDD/Shimadzu, Julius Clinical, Lilly, MagQu, Novartis, Roche Diagnostics, and Siemens Healthineers, and is a co-founder of Brain Biomarker Solutions in Gothenburg AB (BBS), which is a part of the GU Ventures Incubator Program, all outside the submitted work.

The remaining authors declare that the research was conducted in the absence of any commercial or financial relationships that could be construed as a potential conflict of interest.

## Publisher’s note

All claims expressed in this article are solely those of the authors and do not necessarily represent those of their affiliated organizations, or those of the publisher, the editors and the reviewers. Any product that may be evaluated in this article, or claim that may be made by its manufacturer, is not guaranteed or endorsed by the publisher.
