# Supplementary material for: Passive and active suicidal ideation in a population-based sample of older adults: Associations with polygenic risk scores of relevance for suicidal behavior
Source: Front Psychiatry. 2023 Feb 21;14:1101956. doi: 10.3389/fpsyt.2023.1101956 (PMC9989261; doi:10.3389/fpsyt.2023.1101956)
Supplement: Supplementary file 1 [file Table_1.docx]

Supplementary Material

**Supplementary Table 1.** References of the polygenic risk scores employed in the study.

| **Score id** | **Trait** | **Reference** | **Number of individuals in ref-study (discovery + replication sample)** |
| --- | --- | --- | --- |
| Erlangsen_2020 | Suicide attempts | Erlangsen et al. 2020, PMID: 30116032 | 50264 + NA |
| Strawbridge_2019 | Suicidality | Strawbridge et al. 2019, PMID: 30745170 | 120156 + NA |
| gwc-GCST007342 | Depression | Howard et al. 2019, PMID: 30718901 | 446238 + 1306354 |
| gwc-GCST006041 | Major depressive disorder | Hyde et al. 2016, PMID: 27479909 | 326113 + 152127 |
| gwc-GCST007340 | Depressive symptoms | Baselmans et al. 2019, PMID: 30643256 | 1067913 + 228033 |
| ebi-a-GCST005902 | Depression (broad) | Howard et al. 2018, PMID: 29662059 | 322580 + NA |
| ieu-a-1187 | Major depressive disorder | Wray et al. 2018, PMID: 29700475 | 480359 + NA |
| ukb-d-20544_11 | Depression ever diagnosed | https://gwas.mrcieu.ac.uk/datasets/ukb-d-20544_11/ | 117782 + NA |
| Kunkle_2019 | Alzheimer's disease | Kunkle et al. 2019, PMID: 30820047 | 82771 |
| ebi-a-GCST006572 | Cognitive performance | Lee et al. 2018, PMID: 30038396 | 257841 + NA |
| gwc-GCST006442 | Educational attainment | Lee et al. 2018, PMID: 30038396 | 1131881 + NA |
| ebi-a-GCST006940 | Neuroticism | Nagel et al. 2018, PMID: 29500382 | 380506 + NA |
| ebi-a-GCST005232 | Neuroticism | Luciano et al. 2018, PMID: 29255261 | 329821 + 122867 |
| ukb-b-4630 | Neuroticism score | https://gwas.mrcieu.ac.uk/datasets/ukb-b-4630/ | 374323 + NA |
| ukb-b-8476 | Loneliness, isolation | https://gwas.mrcieu.ac.uk/datasets/ukb-b-8476/ | 455364 + NA |
| ebi-a-GCST006942 | Feeling lonely | Nagel et al. 2018, PMID: 29500382 | 376352 + NA |
| gwc-GCST006923 | Loneliness | Day et al. 2018, PMID: 29970889 | 445024 + NA |
| ebi-a-GCST005843 | Ischemic stroke | Malik et al. 2018, PMID: 29531354 | 440328 + NA |
| ukb-b-12493 | Hypertension | https://gwas.mrcieu.ac.uk/datasets/ukb-b-12493/ | 463010 + NA |
| ukb-b-14177 | High blood pressure | https://gwas.mrcieu.ac.uk/datasets/ukb-b-14177/ | 461880 + NA |
| ukb-b-1668 | Atherosclerotic heart disease | https://gwas.mrcieu.ac.uk/datasets/ukb-b-1668/ | 463010 + NA |
| ukb-b-8468 | Angina | https://gwas.mrcieu.ac.uk/datasets/ukb-b-8468/ | 461880 + NA |
