# Supplementary material for: Passive and active suicidal ideation in a population-based sample of older adults: Associations with polygenic risk scores of relevance for suicidal behavior
Source: Front Psychiatry. 2023 Feb 21;14:1101956. doi: 10.3389/fpsyt.2023.1101956 (PMC9989261; doi:10.3389/fpsyt.2023.1101956)
Supplement: Supplementary file 2 [file Table_2.docx]

Supplementary Material

**Supplementary Table 2.** Associations between polygenic risk scores and suicidal feelings based on Paykel questions 1 and 2.

|  |  |  | **Major depression included** | | | | | **Major depression excluded** | | | | |
| --- | --- | --- | --- | --- | --- | --- | --- | --- | --- | --- | --- | --- |
|  |  | **GWAS**  **level** |  |  | **95% CI** | |  |  |  | **95% CI** | |  |
| **Trait** | **Score-id** |  | **Beta** | **SE** | **Lower** | **Upper** | **p-value** | **Beta** | **SE** | **Lower** | **Upper** | **p-value** |
| Suicide attempts | Erlangsen_2020 | 1e-6 | -0.047 | 0.0528 | -0.151 | 0.056 | 0.4 | -0.055 | 0.0617 | -0.176 | 0.066 | 0.4 |
| Suicidality broad | Strawbridge_2019 | 5e-8 | 0.055 | 0.0571 | -0.057 | 0.167 | 0.3 | 0.062 | 0.0667 | -0.069 | 0.192 | 0.4 |
| Depression | gwc-GCST007342 | 5e-8 | 0.153 | 0.0590 | 0.038 | 0.269 | **0.009*** | 0.159 | 0.0677 | 0.026 | 0.292 | **0.02** |
| Major depressive disorder | gwc-GCST006041 | 5e-8 | 0.148 | 0.0528 | 0.045 | 0.252 | **0.005*** | 0.143 | 0.0580 | 0.029 | 0.257 | **0.01*** |
| Depressive symptoms | gwc-GCST007340 | 5e-8 | 0.107 | 0.0598 | -0.010 | 0.224 | 0.07 | 0.106 | 0.0687 | -0.029 | 0.241 | 0.1 |
| Depression (broad) | ebi-a-GCST005902 | 1e-5 | 0.164 | 0.0698 | 0.027 | 0.301 | **0.02** | 0.125 | 0.0809 | -0.033 | 0.284 | 0.1 |
| Depression (broad) | ebi-a-GCST005902 | 5e-8 | 0.074 | 0.1025 | -0.127 | 0.275 | 0.5 | 0.096 | 0.1176 | -0.134 | 0.327 | 0.4 |
| Major depressive disorder | ieu-a-1187 | 1e-5 | 0.062 | 0.0572 | -0.050 | 0.174 | 0.3 | 0.041 | 0.0646 | -0.086 | 0.168 | 0.5 |
| Major depressive disorder | ieu-a-1187 | 5e-8 | 0.108 | 0.0560 | -0.002 | 0.218 | **0.05** | 0.135 | 0.0654 | 0.007 | 0.264 | **0.04** |
| Depression ever diagnosed | ukb-d-20544_11 | 1e-5 | 0.013 | 0.0885 | -0.160 | 0.187 | 0.9 | 0.072 | 0.1006 | -0.126 | 0.269 | 0.5 |
| Depression ever diagnosed | ukb-d-20544_11 | 5e-8 | -0.045 | 0.1156 | -0.271 | 0.182 | 0.7 | 0.053 | 0.1343 | -0.210 | 0.317 | 0.7 |
| Alzheimer's disease | Kunkle_2019 | 1e-5 | -0.087 | 0.0612 | -0.207 | 0.033 | 0.2 | -0.079 | 0.0700 | -0.216 | 0.058 | 0.3 |
| Alzheimer's disease | Kunkle_2019 | 5e-8 | -0.068 | 0.0629 | -0.191 | 0.055 | 0.3 | -0.049 | 0.0717 | -0.189 | 0.092 | 0.5 |
| Cognitive performance | ebi-a-GCST006572 | 1e-5 | -0.200 | 0.0586 | -0.315 | -0.086 | **0.0006*** | -0.223 | 0.0662 | -0.353 | -0.094 | **0.0007*** |
| Cognitive performance | ebi-a-GCST006572 | 5e-8 | -0.169 | 0.0592 | -0.285 | -0.053 | **0.004*** | -0.164 | 0.0680 | -0.297 | -0.031 | **0.02** |
| Educational attainment | gwc-GCST006442 | 5e-8 | -0.007 | 0.0529 | -0.111 | 0.097 | 0.9 | 0.025 | 0.0599 | -0.092 | 0.143 | 0.7 |
| Neuroticism | ebi-a-GCST006940 | 1e-5 | 0.157 | 0.0570 | 0.046 | 0.269 | **0.006*** | 0.157 | 0.0620 | 0.035 | 0.278 | **0.01*** |
| Neuroticism | ebi-a-GCST006940 | 5e-8 | 0.145 | 0.0556 | 0.036 | 0.254 | **0.009*** | 0.178 | 0.0615 | 0.058 | 0.299 | **0.004*** |
| Neuroticism | ebi-a-GCST005232 | 1e-5 | 0.121 | 0.0655 | -0.007 | 0.250 | 0.06 | 0.132 | 0.0743 | -0.014 | 0.277 | 0.08 |
| Neuroticism | ebi-a-GCST005232 | 5e-8 | 0.110 | 0.0737 | -0.034 | 0.254 | 0.1 | 0.157 | 0.0839 | -0.007 | 0.322 | 0.06 |
| Neuroticism score | ukb-b-4630 | 1e-5 | 0.125 | 0.0607 | 0.007 | 0.244 | **0.04** | 0.102 | 0.0667 | -0.028 | 0.233 | 0.1 |
| Neuroticism score | ukb-b-4630 | 5e-8 | 0.111 | 0.0649 | -0.016 | 0.238 | 0.09 | 0.145 | 0.0719 | 0.004 | 0.286 | **0.04** |
| Loneliness, isolation | ukb-b-8476 | 1e-5 | 0.040 | 0.0539 | -0.065 | 0.146 | 0.5 | 0.034 | 0.0639 | -0.091 | 0.159 | 0.6 |
| Loneliness, isolation | ukb-b-8476 | 5e-8 | -0.057 | 0.0532 | -0.161 | 0.047 | 0.3 | -0.052 | 0.0633 | -0.176 | 0.072 | 0.4 |
| Feeling lonely | ebi-a-GCST006942 | 1e-5 | 0.067 | 0.0524 | -0.036 | 0.170 | 0.2 | 0.066 | 0.0599 | -0.052 | 0.183 | 0.3 |
| Feeling lonely | ebi-a-GCST006942 | 5e-8 | 0.026 | 0.0525 | -0.077 | 0.128 | 0.6 | 0.011 | 0.0597 | -0.106 | 0.128 | 0.9 |
| Loneliness | gwc-GCST006923 | 5e-8 | -0.025 | 0.0532 | -0.129 | 0.079 | 0.6 | -0.039 | 0.0610 | -0.159 | 0.080 | 0.5 |
| Ischemic stroke | ebi-a-GCST005843 | 1e-5 | -0.024 | 0.0545 | -0.131 | 0.083 | 0.7 | 0.018 | 0.0628 | -0.105 | 0.142 | 0.8 |
| Ischemic stroke | ebi-a-GCST005843 | 5e-8 | 0.005 | 0.0535 | -0.099 | 0.110 | 0.9 | 0.049 | 0.0618 | -0.072 | 0.170 | 0.4 |
| hypertension | ukb-b-12493 | 1e-5 | -0.013 | 0.0591 | -0.129 | 0.103 | 0.8 | 0.022 | 0.0677 | -0.110 | 0.155 | 0.7 |
| hypertension | ukb-b-12493 | 5e-8 | 0.019 | 0.0550 | -0.089 | 0.126 | 0.7 | 0.104 | 0.0625 | -0.019 | 0.227 | 0.1 |
| High blood pressure | ukb-b-14177 | 1e-5 | 0.087 | 0.0578 | -0.026 | 0.200 | 0.1 | 0.104 | 0.0625 | -0.019 | 0.227 | 0.06 |
| High blood pressure | ukb-b-14177 | 5e-8 | 0.036 | 0.0584 | -0.078 | 0.150 | 0.5 | 0.055 | 0.0671 | -0.077 | 0.186 | 0.4 |
| Atheroscelrotic heart disease | ukb-b-1668 | 1e-5 | -0.015 | 0.0522 | -0.117 | 0.088 | 0.8 | 0.044 | 0.0591 | -0.072 | 0.159 | 0.5 |
| Atheroscelrotic heart disease | ukb-b-1668 | 5e-8 | -0.001 | 0.0548 | -0.108 | 0.107 | 0.99 | 0.067 | 0.0628 | -0.056 | 0.190 | 0.3 |
| Angina | ukb-b-8468 | 1e-5 | 0.079 | 0.0555 | -0.030 | 0.187 | 0.2 | 0.104 | 0.0640 | -0.021 | 0.230 | 0.1 |
| Angina | ukb-b-8468 | 5e-8 | 0.078 | 0.0546 | -0.029 | 0.185 | 0.2 | 0.127 | 0.0641 | 0.002 | 0.253 | **0.05** |

Significant p-values at the level 0.05 are shown in bold. *p-values significant after Bonferroni correction for multiple testing (corrected p-value threshold = 0.01; based on testing of PRSs within five different domains related to suicidal feelings in old age: psychiatric disease, personality, cognitive function/performance, loneliness, vascular disease.
