# Supplementary figures and images for: Passive and active suicidal ideation in a population-based sample of older adults: Associations with polygenic risk scores of relevance for suicidal behavior
Source: Front Psychiatry. 2023 Feb 21;14:1101956. doi: 10.3389/fpsyt.2023.1101956 (PMC9989261; doi:10.3389/fpsyt.2023.1101956)

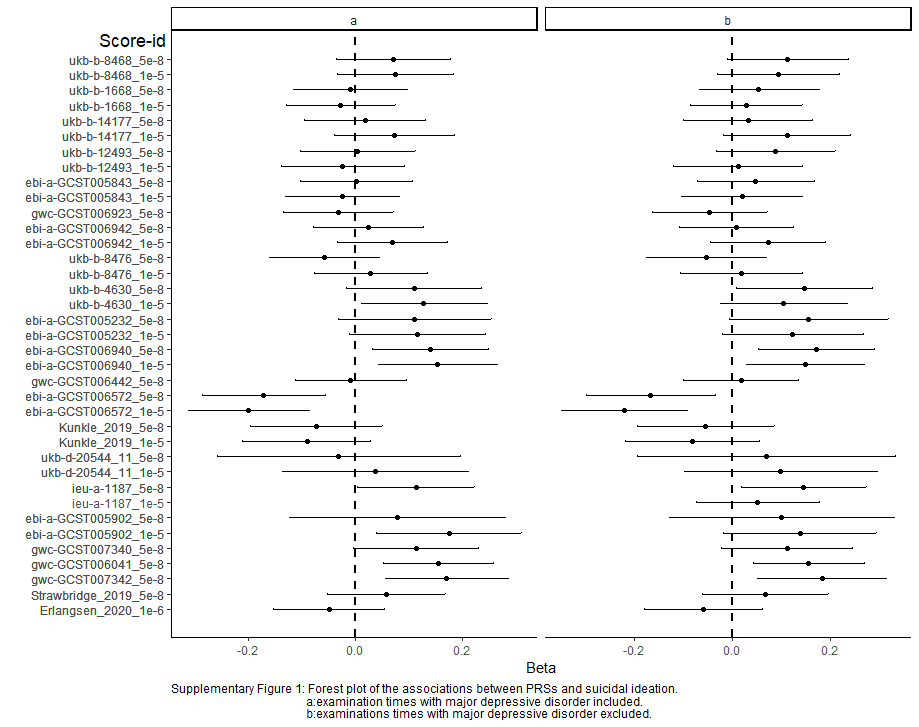

Supplement: Supplementary file 3 [file Image_1.tiff]
